# Association Between Carotid Artery Function and Structure in the Northern Manhattan Study

**DOI:** 10.3389/fneur.2018.00246

**Published:** 2018-04-16

**Authors:** David Della-Morte, Hannah Gardener, Chuanhui Dong, Matthew Markert, Digna Cabral, Mitchell S. V. Elkind, Ralph L. Sacco, Tatjana Rundek

**Affiliations:** ^1^Department of Neurology, Miller School of Medicine, University of Miami, Miami, FL, United States; ^2^Department of Systems Medicine, School of Medicine, University of Rome Tor Vergata, Rome, Italy; ^3^Department of Human Sciences and Quality of Life Promotion, San Raffaele Roma Open University, Rome, Italy; ^4^Department of Neurology, Kansas University Medical Center, Kansas City, MO, United States; ^5^Division of Stroke, Department of Neurology, Columbia University, New York, NY, United States

**Keywords:** carotid artery, carotid intima media thickness, stiffness, atherosclerosis, carotid plaque, carotid ultrasound, arterial remodeling

## Abstract

**Background and purpose:**

Carotid plaque (CP), carotid intima media thickness (cIMT), and stiffness (STIFF) are pre-clinical markers of atherosclerosis and predictors of cerebrovascular disease (CVD). We sought to investigate whether STIFF is a significant determinant of cIMT and CP, which may provide an insight into the mechanism by which STIFF adds to the risk of CVD.

**Methods:**

We analyzed 876 stroke-free subjects from the Northern Manhattan Study with available ultrasound measures. To obtain the associations with STIFF, we performed multivariable-adjusted regression, negative binomial regression (for CP number), and multinomial logistic regression (for plaque area).

**Results:**

The mean age was 64 ± 9 years; 63% women and 65% Caribbean Hispanics. The mean cIMT was 0.93 ± 0.9 mm, the mean diastolic diameter 6.24 ± 0.94 mm, and STIFF 8.6 ± 6.2 ln mmHg. Prevalence of CP was 57%, and the mean total plaque area was 22.6 ± 23.0 mm^2^. STIFF was positively associated with cIMT but not with CP. There was an association between diastolic diameter and thick plaque. For each millimeter increase in diastolic diameter, there was about a 20% increased risk of having thick plaque (vs. no plaque). In longitudinal analyses, each millimeter increase in diastolic diameter was associated with a 37% increased risk of incident plaque.

**Conclusion:**

Increased STIFF was associated with increased cIMT and carotid artery dilatation with greater plaque burden. Increased cIMT and plaque burden represent vascular remodeling likely resulting from the two different age-related mechanisms, one that includes diffuse wall thickening (cIMT) with STIFF and another that incorporates focal atherosclerosis (plaque) with luminal dilatation.

## Introduction

Atherosclerosis is the underlying process of most cardiovascular disease (CVD) (1) leading to luminal stenosis with flow restriction or to plaque rupture (2). Carotid plaque (CP), carotid intima media thickness (cIMT), and arterial stiffness (STIFF) are well-established subclinical markers of atherosclerosis and significant predictors of CVD (2, 3). They are biologically and genetically distinct phenotypes of atherosclerosis (4, 5). CP thickness and area assessed by ultrasound are direct measures of atherosclerotic plaque burden (6) and better predictors of CVD than cIMT (4, 7, 8).

Stiffness is a functional measure of the arterial wall’s resistance to pressure deformation during the cardiac cycle (9). STIFF and arterial dilatation result from a degenerative process affecting mainly the extracellular matrix of elastic arteries where the principal risk factor is aging. Arterial dilatation and STIFF may be early markers of structural atherosclerotic changes and potentially targeted for early anti-atherosclerotic interventions (10). However, information on the relationship between arterial wall function and structure in the general population is sparse. We sought to investigate these associations in a stroke-free population from the Northern Manhattan Study (NOMAS).

## Materials and Methods

### Study Population

Northern Manhattan Study is a population-based study designed to determine the incidence of stroke and CVD described previously (11). A total of 3,298 subjects were enrolled. As a part of the Carotid Imaging Study (2), 876 individuals with available ultrasonographic measurements and signed written informed consents in accordance with the Declaration of Helsinki were included in analyses. NOMAS was approved by the Institutional Review Boards of Columbia University and the University of Miami.

Data were collected through interviews using standardized collection instruments, review of the medical records, and physical examination (11). Vascular risk factors (vRF) and physical activity were described previously (2, 12).

### Carotid Ultrasound

High-resolution two-dimensional carotid ultrasound imaging (Figure S1 in Supplementary Material) was performed according to the standardized scanning and reading protocols (2). The left and right carotid bifurcations, the internal carotid arteries (ICA), and common carotid arteries (CCA) were imaged (13). Plaque was defined as focal wall thickening or protrusion in the lumen more than 50% greater than the surrounding wall thickness. CP boundaries were traced offline using automatic edge detection system (M’Ath Inc., Paris, France). The sum of plaque areas in all carotid arteries from the both side of the neck was expressed as total plaque area (TPA) in square millimeter. IMT (mm) in all carotid segments was measured in areas free of plaque. cIMT was calculated as a composite measure combining the near and the far walls of CCA IMT, bifurcation IMT, and ICA IMT of both neck sides and examined continuously as a mean of the mean measurements of the 12 sites. The offline measurement of STIFF was performed as described (14). STIFF (ln mmHg) was calculated as [(the natural log transformation of (systolic BP − diastolic BP))/strain], where strain was [(systolic diameter − diastolic diameter)/diastolic diameter].

A subgroup of 267 individuals with two carotid ultrasound images apart were included in an analysis of the relationship between DD, STIFF, and strain with incident plaques and the change in the maximum plaque thickness and cIMT. Incident plaque was defined as an increase in the number of plaques between carotid ultrasound measurements, and the change in maximal plaque thickness and cIMT was defined as the thickness at follow-up minus the thickness at baseline.

## Statistical Analysis

In cross-sectional analyses, independent variables were DD, strain, and STIFF and each examined continuously. The dependent variables were the mean cIMT, plaque number, plaque thickness, and TPA. TPA was examined in three categories: no plaque and tertiles of TPA distribution (tertile 1–2 and the top tertile). Plaque thickness was also examined in three categories: no plaque, plaque < 1.9 mm, and plaque > 1.9 mm. The latter cutoff was used to define thick plaque as it has been significantly associated with clinical outcomes in this cohort (2). Multinomial logistic regression was used to examine the associations of DD, strain, and STIFF with plaque thickness and TPA, with no plaque as the reference. Due to overdispersion of plaque number, negative binomial regression was used to examine the associations with plaque number, examined continuously as dependent variable. cIMT was examined as a continuous outcome using linear regression. We used a sequence of multivariable-adjusted regression models. The first model controlled for demographic variables (age, sex, and race/ethnicity), the second model controlled for demographics and anti-hypertensive medication, and the third model additionally controlled for current smoking, diabetes, moderate alcohol use, moderate to heavy physical activity, BMI, and hypercholesterolemia. We did not adjust for hypertension in order to avoid overadjustment as STIFF is a metric that includes systolic and diastolic BP measurements. We examined potential effect modification by demographic variables, diabetes, smoking, and lipids, using interaction terms in the third model.

An exploratory prospective analysis was conducted for strain, STIFF, and DD in association with incident plaque using logistic regression and with the change in maximal plaque thickness and cIMT using linear regression models. The same three models described earlier were used, additionally controlling for the time span between carotid measurements.

## Results

Among 876 subjects (mean age 64 ± 9 years), 63% were women, 65% Caribbean Hispanic, 17% black, and 16% white. The mean cIMT was 0.93 ± 0.9 mm, the mean STIFF 8.6 ± 6.2 ln mmHg (median = 6.9, range = 1.6–51.5), the mean strain 0.08 ± 0.04 (median = 0.07, range = 0.01–0.30), and the mean DD 6.24 ± 0.94 mm (median = 6.10, range = 3.90–10.50). Prevalence of CP was 57% (plaque number distribution: 1 plaque = 19%, 2 = 17%, 3 = 9%, 4 = 5%, 5 = 4%, 6 = 2%, 7 = 1%, 8 = < 1%, and 9 = < 1%), and 38% had plaque > 1.9 mm. Among those with plaque, the mean TPA was 22.6 (±23.0) mm^2^, median = 14.8 mm^2^. TPA distribution for the first two tertiles (*N* = 326) ranged from 2.2 to 21.6 mm^2^ and for the third tertile (*N* = 176) from 21.7 to 168.8 mm^2^. Table 1 shows the covariate characteristics of the study population overall and by TPA categories.

**Table 1:** Characteristics of the study population.

Variable	Study population *N* = 876	No plaque *N* = 374	Plaque area tertiles 1–2*N* = 326	Plaque area tertile 3*N* = 176
Age, mean ± SD	64 ± 9	61 ± 8	65 ± 9	69 ± 8
Male sex, *N* (%)	328 (37)	136 (36)	118 (36)	74 (42)
**Race/ethnicity, *N* (%)**				
Black	149 (17)	52 (14)	58 (18)	39 (22)
White	144 (16)	45 (12)	53 (16)	46 (26)
Hispanic	568 (65)	269 (72)	210 (64)	89 (51)
Current smoker, *N* (%)	141 (16)	54 (14)	52 (16)	35 (20)
Moderate alcohol use, *N* (%)	367 (42)	160 (43)	139 (43)	68 (39)
Moderate-heavy physical activity, *N* (%)	100 (12)	43 (12)	36 (11)	21 (12)
Diabetes, *N* (%)	157 (18)	51 (14)	59 (18)	47 (27)
Anti-hypertensive medication use, *N* (%)	346 (40)	140 (38)	115 (35)	91 (52)
Hypercholesterolemia, *N* (%)	538 (61)	222 (59)	198 (61)	118 (67)
BMI (kg/m^2^), mean ± SD	28.61 ± 5.29	29.97 ± 5.47	28.42 ± 4.93	28.19 ± 5.50
Stiffness (ln mmHg), mean ± SD	8.61 ± 6.17	7.95 ± 5.47	8.59 ± 6.14	10.08 ± 7.32
Strain, mean ± SD	0.08 ± 0.04	0.08 ± 0.04	0.08 ± 0.04	0.08 ± 0.04
Diastolic diameter (mm), mean ± SD	6.24 ± 0.94	6.15 ± 0.90	6.24 ± 0.91	6.45 ± 1.02
Carotid intima media thickness (mm), mean ± SD	0.93 ± 0.09	0.91 ± 0.08	0.93 ± 0.09	0.99 ± 0.10

In univariate analysis, significant association was present between age and STIFF (*p* < 0.0001), DD (*p* < 0.0001), and strain (*p* = 0.0003). Table 2 shows the relationship between DD and STIFF with cIMT and the plaque phenotypes and in the sequence of multivariable-adjusted models. DD and STIFF were both positively associated with cIMT in all three models. For the plaque phenotypes, the only association observed was a positive association between DD and thick plaque (>1.9 mm). Each millimeter increase in DD was associated with a 20% increased risk of thick plaque (vs. no plaque). Strain and STIFF were not associated with plaque thickness. DD and STIFF were not associated with plaque number neither with plaque area. No significant effect modifications were observed.

**Table 2:** Association of carotid stiffness and diastolic diameter with carotid intima media thickness (cIMT) and plaque phenotypes.

	Estimate, *p*-value		OR (95% CI)			
	cIMT (mean of the max)	Plaque number (continuous)	Plaque thickness < 1.9 mm vs. no plaque	Plaque thickness > 1.9 mm vs. no plaque	Plaque area tertiles 1–2 vs. no plaque	Plaque area tertile 3 vs. no plaque
**Diastolic diameter, per 1 unit**						
Model 1 Model 2 Model 3	**0.019 (<0.0001)** **0.019 (<0.0001)** **0.019 (<0.0001)**	0.053 (0.22) 0.049 (0.27) 0.036 (0.41)	0.83 (0.66–1.03) 0.84 (0.68–1.06) 0.84 (0.67–1.06)	**1.22 (1.03–1.46)** **1.23 (1.03–1.46)** **1.21 (1.01–1.46)**	1.03 (0.87–1.22) 1.06 (0.89–1.26) 1.06 (0.88–1.27)	1.18 (0.96–1.45) 1.14 (0.92–1.41) 1.10 (0.88–1.38)

**Stiffness, per 1 unit**						
Model 1 Model 2 Model 3	**0.001 (0.03)** **0.001 (0.03)** **0.001 (0.05)**	0.010 (0.10) 0.010 (0.13) 0.009 (0.14)	1.00 (0.97–1.04) 1.01 (0.97–1.04) 1.01 (0.97–1.04)	1.02 (0.99–1.04) 1.02 (0.99–1.04) 1.02 (0.99–1.04)	1.00 (0.98–1.03) 1.01 (0.98–1.03) 1.01 (0.98–1.03)	1.03 (1.00–1.06) 1.02 (0.99–1.06) 1.02 (0.99–1.06)

*Model 1: controlling for demographics (age, sex, and race/ethnicity)*.

*Model 2: controlling for demographics and anti-hypertensive medication use*.

*Model 3: controlling for demographics, anti-hypertensive medication use, smoking, diabetes, hypercholesterolemia, moderate alcohol use, moderate-heavy physical activity, and BMI*.

*Significant associations and corresponding *p* values (*p* < 0.05) are in bold to aid the viewer*.

In a longitudinal analysis, 115 individuals had a new plaque at follow-up, including 30 new plaques among 101 without plaque at baseline. The mean time span between measurements was 3.2 years (range = 2.8–5.4), the mean change in maximal plaque thickness was 0.35 ± 1.07 mm, and the mean change in cIMT was 0.16 ± 0.15 mm. In model 1, STIFF was not associated with incident plaque (data not shown), but DD was positively associated with incident plaque in model 1 (OR = 1.36, 95% CI = 1.01–1.82) and model 3 (OR = 1.37, 95% CI = 1.00–1.89). There was a suggested positive association of DD with maximum plaque thickness (model 1, beta = 0.15, *p* = 0.05; model 3 beta = 0.15, *p* = 0.06). No association of DD nor STIFF was found with the change in cIMT.

## Discussion

In our elderly community cohort, we report a significant association between increased STIFF and cIMT and between larger carotid diameter and CP burden. These associations were independent of demographics and major vRF, directly linking parameters of arterial function and structure through the arterial wall remodeling processes. Our findings may help to better understand the link between arterial wall mechanics and arterial remodeling in early atherosclerosis.

The associations of STIFF with cIMT and plaques have been reported previously (10, 15). In the Atherosclerosis Risk in Communities, the positive association between STIFF and cIMT was found only in the thickest cIMT that represented 10% of the carotids, likely representing plaque. This relationship was explained by the presence of endothelial damage enhanced by intraluminal stress in STIFF arteries and accumulation of atherosclerotic material in arterial wall. However, the mechanisms of arterial remodeling leading to increased cIMT in contrast to arterial plaque may be different and remains largely unexplained. Our study may suggest different remodeling mechanisms leading to either diffuse arterial wall thickness (cIMT) that is largely dependent on arterial STIFF or to focal atherosclerotic changes (plaque) dependent on luminal dilatation. According to the Glagov’s vascular remodeling mechanism, arteries remodel to maintain constant flow despite increases in atherosclerotic lesion mass (16). In an experimental model of the carotid arteries in rabbits, flow-induced arterial dilatation was accompanied by an adaptive remodeling of carotid intima (17), suggesting that arterial dilatation is an early marker of atherosclerotic lesion.

Traditional and less-traditional vRF impact arterial remodeling and contribute to the stiffening of the arterial tree (14, 18, 19). In our study, the association between STIFF and cIMT was not modified by vRF, suggesting a direct flow-pressure contribution to the wall injury that deserves further exploration.

We found a positive association between DD and thick plaque. A study conducted in hypertensive patients demonstrated that change in diameter of CCA directly impacted plaque thickness, supporting our findings (20). Based on the Glagov’s remodeling mechanism (21), the enlargement of the DD may preferentially induce one phenotype of atherosclerosis, i.e., plaque, over another, i.e., cIMT (22, 23). However, the mechanism leading to the preferential atherosclerotic phenotype remains unclear.

In our longitudinal analysis, we found independent associations between enlargement of DD and incident plaque and increased maximum plaque thickness. Conversely, no associations were found for STIFF with plaque and cIMT and for DD and cIMT. In support, the Plaque At RISK study (24) has demonstrated a stronger association for DD, plaque progression, and CVD than for cIMT. These results suggest that plaque development may be more affected by the arterial remodeling mechanism than cIMT. Driving forces that induce plaque progressing with aging may be associated with the lumen dilatation as an adaptive response to increased pressure in the arteries (25). Moreover, a significant interaction between plaque and STIFF is present only in advanced stage of the atherosclerotic lesion (26). Larger longitudinal studies with multiple follow-up ultrasound measurements are needed to establish temporal associations between these phenotypes of atherosclerosis.

A lack of the association between STIFF and plaque in our study is in contrast to the findings from the Rotterdam Study (15), where this correlation was present. The discrepancy may be related to different study populations, ultrasound methodologies, and the measurement of STIFF (carotid ultrasound vs. pulse wave velocity), which depends by several parameters and may not be accurate. Local measurement of STIFF by ultrasound evaluation of arterial distensibility and compliance is able to a better evaluation of the absolute or relative changes in the arterial volume and distending pressure.

Various studies have adopted different plaque and cIMT definitions (27). Our cIMT method is based on the Mannheim consensus to avoid discrepancies reported in major cIMT studies (28). Development of novel methods such as non-invasive vascular ultrasound elastography may provide more direct measurements of the arterial walls mechanical properties at the level of plaque with increased accuracy and validity. Strengths of the current study include a population-based design, inclusion of both cross-sectional and prospective analyses, and the use of multiple plaque phenotypes for comparisons. The major limitation is that our study cohort is old and results may not be generalizable to younger populations. The prospective analysis had limited power and as in any epidemiology study, confounding is a possibility, and causality cannot be inferred.

## Ethics Statement

As a part of the Carotid Imaging Study (26), 876 individuals with available ultrasonographic measurements and signed written informed consents in accordance with the Declaration of Helsinki were included in analyses. NOMAS was approved by the Institutional Review Boards of Columbia University and the University of Miami.

## Author Contributions

DD-M: conceived the presented idea, conceived and planned the analysis of the data, contributed to the interpretation of the results, and wrote the manuscript. HG: performed statistical analysis, contributed to the interpretation of the results, and made a critical revision of the manuscript. CD: performed statistical analysis, contributed to the interpretation of the results, and made a critical revision of the manuscript. MM: contributed to the interpretation of the results and made a critical revision of the manuscript. DC: collected the data for the analysis. ME: contributed to the interpretation of the results and made a critical revision of the manuscript. RS: handled funds, conceived the presented idea, conceived and planned the analysis of the data, contributed to the interpretation of the results, and made a critical revision of the manuscript. TR: handled funds, conceived the presented idea, conceived and planned the analysis of the data, contributed to the interpretation of the results, and wrote the manuscript.

## Conflict of Interest Statement

The authors declare that the research was conducted in the absence of any commercial or financial relationships that could be construed as a potential conflict of interest.

## Supplementary Material

Figure S1 Carotid Ultrasound procedures. High-resolution two-dimensional carotid ultrasound imaging was performed to measure carotid function and structure in 876 subjects from the Northern Manhattan Study (NOMAS). BP, blood pressure; CCA, common carotid arteries; cIMT, carotid intima media thickness; DBP, diastolic blood pressure; DD, diastolic diameter; ECA, external carotid arteries; ICA, internal carotid arteries; SBP, systolic blood pressure; DD, diastolic diameter; SD, systolic diameter; TPA, total plaque area (mm^2^).
