# Supplementary figures and images for: Association Between Carotid Artery Function and Structure in the Northern Manhattan Study
Source: Front Neurol. 2018 Apr 16;9:246. doi: 10.3389/fneur.2018.00246 (PMC5911635; doi:10.3389/fneur.2018.00246)

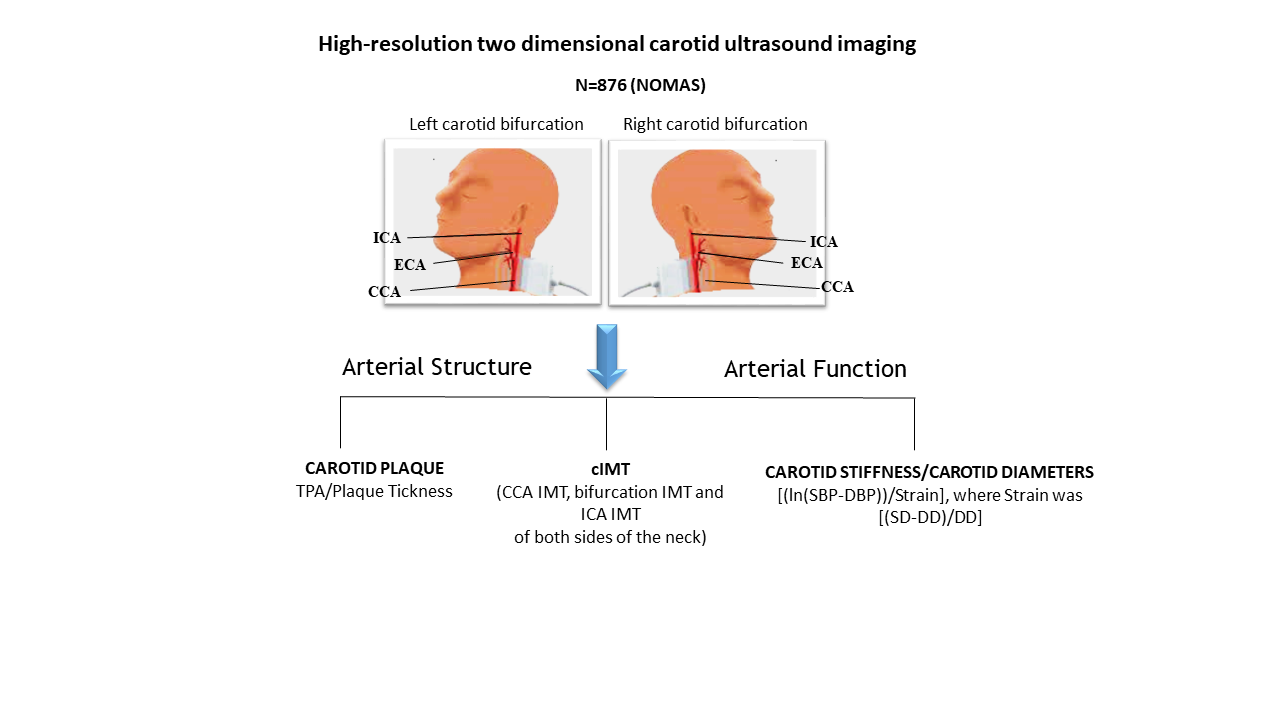

Supplement: Figure S1 — Carotid Ultrasound procedures. High-resolution two-dimensional carotid ultrasound imaging was performed to measure carotid function and structure in 876 subjects from the Northern Manhattan Study (NOMAS). BP, blood pressure; CCA, common carotid arteries; cIMT, carotid intima media thickness; DBP, diastolic blood pressure; DD, diastolic diameter; ECA, external carotid arteries; ICA, internal carotid arteries; SBP, systolic blood pressure; DD, diastolic diameter; SD, systolic diameter; TPA, total plaque area (mm2). [file image_1.tif]
